# How to prove the existence of metabolons?

**DOI:** 10.1007/s11101-017-9509-1

**Published:** 2017-04-26

**Authors:** Jean-Etienne Bassard, Barbara Ann Halkier

**Affiliations:** 1 Plant Biochemistry Laboratory, Center for Synthetic Biology, VILLUM Research Center “Plant Plasticity”, Department of Plant and Environmental Sciences, University of Copenhagen, Copenhagen, Denmark; 2 DynaMo Center, Department of Plant and Environmental Sciences, University of Copenhagen, Copenhagen, Denmark

**Keywords:** Fluorescence-based protein–protein interaction, Fluorescence correlation spectroscopy, Fluorescence lifetime imaging microscopy, Yeast-2-hybrid screen

## Abstract

Sequential enzymes in biosynthetic pathways are organized in metabolons. It is challenging to provide experimental evidence for the existence of metabolons as biosynthetic pathways are composed of highly dynamic protein–protein interactions. Many different methods are being applied, each with strengths and weaknesses. We will present and evaluate several techniques that have been applied in providing evidence for the orchestration of the biosynthetic pathways of cyanogenic glucosides and glucosinolates in metabolons. These evolutionarily related pathways have ER-localized cytochromes P450 that are proposed to function as anchoring site for assembly of the enzymes into metabolons. Additionally, we have included commonly used techniques, even though they have not been used (yet) on these two pathways. In the review, special attention will be given to less-exploited fluorescence-based methods such as FCS and FLIM. Ultimately, understanding the orchestration of biosynthetic pathways may contribute to successful engineering in heterologous hosts.

## Introduction

Plants are sessile organisms and their survival is dependent on unique specialized biosynthetic capacities, which require a high degree of functional organization and infrastructure. Cellular processes have been suggested to be organized through compartmentalization and assembly of multi-enzyme complexes in metabolons (Winkel 2004; Jørgensen et al. 2005). In this last decade, several studies proposed formation of metabolons in diverse metabolic pathways from plants to animals. For example, metabolons involved in lignin biosynthesis (Chen et al. 2011; Bassard et al. 2012), sporopollenin biosynthesis (Lallemand et al. 2013), photosynthetic complex (Szecowka et al. 2013), flavonoid biosynthesis (Crosby et al. 2011; Dastmalchi et al. 2016), fatty acid biosynthesis (Kwiatkowska et al. 2015), dhurrin pathway (Nielsen et al. 2008; Laursen et al. 2016), Krebs cycle (Wu and Minteer 2015), cholesterol synthesis (Luu et al. 2015), and purine synthesis (An et al. 2008; Kyoung et al. 2015). Metabolons have been described as supramolecular complexes of sequential metabolic enzymes and cellular structural elements (Srere 1985). This definition is still valid, but recent advances highlight new characteristics of these organizations. Accordingly, metabolon definition could be extended to “Transient and dynamic supramolecular organization of cooperating, often consecutive enzymes of a metabolic pathway, which often is associated with structural elements of the cell (e.g. membrane, cytoskeleton) and non-enzymatic proteins. Metabolon components can be specific to one metabolon or dynamically shared with other metabolons for swift adaptation of the metabolite profile to environmental changes, challenges and cellular needs”. The organization of metabolic pathways at the molecular level is expected to have several advantages, such as to increase local concentrations of the enzymes and their substrates, to improve channeling of intermediates into specific sub-pathways and to increase metabolic fluxes and sequestration of reactive intermediates (Jørgensen et al. 2005; Ralston and Yu 2006; Laursen et al. 2015). The swift adaptation of plant metabolism to environmental challenges has been proposed to specifically result from transient and dynamic metabolon formations (Narayanaswamy et al. 2009; Møller 2010; Kyoung et al. 2015; Laursen et al. 2016; Dastmalchi and Facchini 2006). Within the crowded intracellular environment, proteins are constantly coming into physical contact. There is diversity in duration, specificity and frequency of these interactions (Marsh and Teichmann 2015). Dynamic co-clustering of enzymes in compact repetitive agglomerates was suggested to accelerate processing of intermediates (Castellana et al. 2014). Membrane-bound proteins, such as cytochromes P450 (P450), may serve as nucleation factors for the assembly of metabolons at the Endoplasmic Reticulum (ER) membrane surface (Winkel 2004; Jørgensen et al. 2005; Ralston and Yu 2006; Bassard et al. 2012). The dynamic and transient nature of the interactions between enzymes in a biosynthetic pathway has made it difficult to prove that metabolons exist.

The biosynthetic pathways of the evolutionarily related amino acid-derived cyanogenic glucosides and glucosinolates have been proposed to form metabolons as both pathways have unstable intermediates that spontaneously would result in abortion of the pathways, if the next enzyme was not in close proximity to the previous enzyme. Multiple approaches have been undertaken to provide experimental evidence for metabolons. Here, we will describe the evidence for the metabolon hypothesis using the cyanogenic dhurrin pathway in *Sorghum bicolor* and the glucosinolate pathway in *Arabidopsis thaliana* as case studies. The cyanogenic dhurrin is formed from tyrosine in a pathway catalyzed by two ER-anchored cytochromes P450, sequentially CYP79A1 and CYP71E1, and one soluble enzyme UGT85B1 and a NADPH-cytochrome P450-reductase supporting the P450s (Laursen et al. 2016). The biosynthetic pathway of the glucosinolate structure consists of seven soluble or ER-anchored enzymes (CYP79, CYP83, GST, GGP1, C-S lyase, UGT, SOT) and a P450-supporting NADPH-cytochrome P450-reductase (Sønderby et al. 2010).

In this review, we present the in vitro and in vivo techniques that have been applied to provide experimental evidence for the existence of metabolons for these two pathways. In addition, we include techniques that are commonly used, even though they have not been used on these two pathways. The review covers methods including yeast-2-hybrid (Y2H), co-immunoprecipitation (Co-IP), tandem affinity purification (TAP), bimolecular fluorescence complementation (BiFC), fluorescence correlation spectroscopy (FCS), and the fluorescence/förster resonance energy transfer (FRET)-based techniques including acceptor photobleaching FRET, sensitized FRET, and fluorescence lifetime imaging microscopy (FLIM). Most of these techniques have been widely used and are under perpetual refinements. A PubMed search revealed that the most popular techniques are the Y2H and FRET-based techniques (Table 1). We will present the principle of each technique, and discuss strengths and weaknesses for the use of each of them. Particular attention will be given to the FCS and FLIM techniques as we regard these techniques very powerful but underexploited *in planta*.

**Table 1:** Comparison of the number of references on different protein–protein interaction techniques searched for in PubMed

Pubmed queries	Total Pubmed occurrences	Pubmed occurrences associated to plant
“fluorescence resonance energy transfer” NOT “fluorescence lifetime imaging microscopy”	12,850	309
“yeast two hybrid”	10,583	1661
Co-immunoprecipitation	7529	290
“bimolecular fluorescence complementation”	1024	546
“fluorescence correlation spectroscopy”	2145	30
“fluorescence lifetime imaging microscopy”	722	44
“tandem affinity purification”	676	77

Pubmed occurrences about the different techniques presented in this review. Pubmed (https://www.ncbi.nlm.nih.gov/pubmed) queried towards the end of October 2016

## Yeast-2-hybrid methods

Probably the most widely used technique to detect protein–protein interaction is the yeast-two-hybrid assay. In the conventional yeast-two hybrid method, protein interactions bring together a DNA-binding domain and a transactivation domain of the GAL4 transcription factor in the nucleus (Fields and Song 1989; Braun et al. 2013). Spurious self-activators in the nucleus often give rise to false positive interactions. To overcome this problem, the split-ubiquitin system was developed (Stagljar et al. 1998). In brief, the split-ubiquitin principle is based on that the ubiquitin protein can be split into two stable moieties, an N-terminal fragment called Nub and a C-terminal fragment called Cub. The wild-type Nub (referred to as NubI) is capable of spontaneous re-association with Cub to form a full-length “pseudo-ubiquitin” molecule. In the mutated NubG fragment the spontaneous association between Nub and Cub is prevented. The split-ubiquitin approach is based on the detection of the in vivo processing of a reconstituted split ubiquitin. The bait protein—which can be either naturally membrane-bound or artificially membrane-anchored—is fused to a Cub moiety linked to a transcription factor, while a prey protein is fused to the NubG fragment. Upon interaction of bait and prey proteins tagged with either Nub and Cub, ubiquitin reconstitution occurs and leads to the proteolytic cleavage and subsequent release of a transcription factor that triggers the activation of a reporter system enabling easy detection. In this manner, the membrane-based yeast-two-hybrid system enables detection of interactions between membrane proteins in a natural environment (Kittanakom et al. 2009; Petschnigg et al. 2012). The split-ubiquitin-based yeast-two-hybrid approach detects both stable and transient binary protein interactions, but has a reputation of generating false positives (Hengen 1997) and also false negative (see under new tools). As the interactions are generated under heterologous conditions, the results must subsequently be validated under physiological conditions.

In the glucosinolate biosynthetic pathway, several split-ubiquitin-based yeast-two-hybrid screens—both targeted and untargeted—have been performed. In a targeted yeast-2-hybrid approach where all the biosynthetic enzymes were used as bait and prey, respectively, only the UGTs and SOTs in the core pathway interacted (Andersen 2012). This indicates that the pen- and ultimate steps in a biosynthesis are likely to contribute to specificity of the pathway. Untargeted yeast-2-hybrid screens using the CYP83A1 (for aliphatic glucosinolates) and CYP83B1 (for indole glucosinolates) as bait identified, respectively, 33 and 27 interacting proteins, of which 6 candidates were found in screens with both baits (Nintemann 2016). The latter included members of a small family of interactors, HR-like lesion-inducing proteins, potentially providing a direct link to defense signaling (Nintemann et al. unpubl. res.). Unexpectedly, none of the proteins was one of the enzymes in the biosynthetic pathway. Although the candidate genes await *in planta* validation, the findings suggest that most of the enzymes are transiently interacting, in a dynamically organized metabolon, which impairs their identification via yeast-2-hybrid. Noticeable, apparent scaffolding proteins and assembly chaperones were absent amongst the candidate genes. The data suggests that rather than viewing the individual steps as part of a robust metabolon with tight physical interactions, the pathway is likely orchestrated as a cluster of enzymes that dynamically may self-assemble stochastically through transient interactions in highly organized cytoplasmic microenvironments.

## Co-immunoprecipitation (Co-IP)

The basic principle for any Co-IP is extracting proteins interacting with a given protein in biological samples by immunoprecipitation, followed by identification of the proteins by proteomics. As the interaction between the proteins has to last throughout the extraction procedure, Co-IP experiments typically report robust interactions. To enable more transient interactions to be reported, crosslinking of the proteins prior to the extraction has successfully been used (Merkley et al. 2013; Chen et al. 2014). Due to the challenge in having specific antibodies against a target of interest, using tags to which commercial antibodies are available has become a common practice. The Green Fluorescent Protein (GFP) fluorophore tag is well-documented to form a self-contained and stable structure independent of its fusion partners and often does not interfere despite its large size (Laursen et al. 2016). Furthermore, transgenic lines with fluorophore-tagged versions of the protein of interest are often generated for localization purposes and therefore available for Co-IP experiments using commercial GFP antibodies (Weis et al. 2013; Speth et al. 2014). As an alternative to use of the classical antibodies, there is now available a promising technique, still not adopted in plant research, which use a distinct type of heavy-chain-only antibodies that in nature is found in sera of camelids (Deffar et al. 2009; Dmitriev et al. 2016). From these antibodies, the smallest intact functional antigen-binding single domain is the VHH fragment (only 15 kD), also known as a nanobody. In a GFP trap, nanobodies directed towards the fluorophore protein is coupled to a matrix (e.g. agarose beads, magnetic agarose beads, magnetic particles) and used for Co-IP of GFP fusion proteins and their interacting partners.

In the glucosinolate pathway, transgenic lines were generated with fluorophore-tagged CYP83A1 and CYP83B1 (Nintemann 2016), that are markers for the aliphatic and indole glucosinolates derived from methionine and tryptophan, respectively. Amongst the protein identified in the Co-IP experiment using the fluorophore-tagged lines in combination with GFP traps, none of the other biosynthetic enzymes in the pathway were identified (Vik and Svozil unpubl. res.). The results are in agreement with the results obtained with the yeast-2-hybrid experiments, i.e. rather than forming a tight metabolon the enzymes may self-assemble stochastically through transient, weak interactions.

## Tandem affinity purification (TAP) method

The technique was developed in 1998 (Rigaut et al. 1999). The first article that mentions the purification of protein complexes from plant via the TAP method was published in 2004 (Rohila et al. 2004). TAP has become one of the most popular methods for purification of in vivo protein complexes and for identification of their components by mass spectrometry (MS), thanks to regular optimizations of the method to filter hits using database of background proteins from different experiments (van Leene et al. 2011; Goossens et al. 2016), the development of several tags (Andrès et al. 2011), the use of mild detergents (e.g. digitonin, dodecylmaltoside, nonidet P-40), and the advent of high-throughput, ultrasensitive MS and protein sequence databases.

**Fig. 1:**
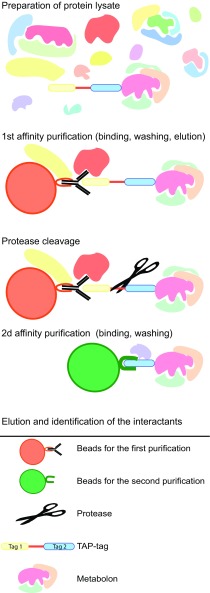
Schematic representation of the tandem affinity purification procedure. The two-step affinity purification protocol involves preparation of the cell lysate, followed by the first affinity purification. Subsequently follows cleavage of the first tag, purification using the second tag, and finally elution of the protein complex to be analyzed by mass spectrometry

The TAP method relies on the application of a two-step affinity purification protocol (Fig. 1). The first tag is a fusion protein containing a strong antigenic region (such as Protein G or GFP) fused to a separate smaller tag (such as streptavidin or calmodulin-binding peptide). The two tags are usually linked by a specific cleavage site (such as the Tobacco Etch Virus or Rhinovirus 3C protease cleavage sites). Details on tag variations already used, their limitations, as well as critical conditions for TAP are available elsewhere (Li 2010; Andrès et al. 2011; van Leene et al. 2011; Gerace and Moazed 2015; Goossens et al. 2016). For the solubilization and isolation of membrane protein complexes, detergent type and detergent concentration should be chosen and tested regarding the tagged-protein to be purified. The two-step purification protocol may wash out partners in weak or transient interactions and thus be problematic for the study of metabolons. The tag may not be exposed to the affinity beads and the protease may in some conditions unspecifically cleave target proteins. Finally, large-scale analysis of the interactome using TAP tagging is time-consuming and expensive.

The TAP method has successfully been used with different plant species and with both membrane-bound and soluble proteins, for example with *M. trunculata* (Goossens et al. 2016), *A. thaliana* (Bassard et al. 2012; van Leene et al. 2016), *O. sativa* (Nallamilli et al. 2013). However, TAP method was never used on our two model pathways. The TAP method allows for identification and quantification of specific native protein complexes or networks, without prior knowledge of complex composition and with a reduced background of contaminating proteins compared to single step purification methods (co-immunoprecipitation or pull-down techniques). The TAP method is robust and simple when studying stable protein complexes and it is possible to fine tune the purification stringency by adjusting washing steps and buffers.

## Bimolecular fluorescence complementation (BiFC)

**Fig. 2:**
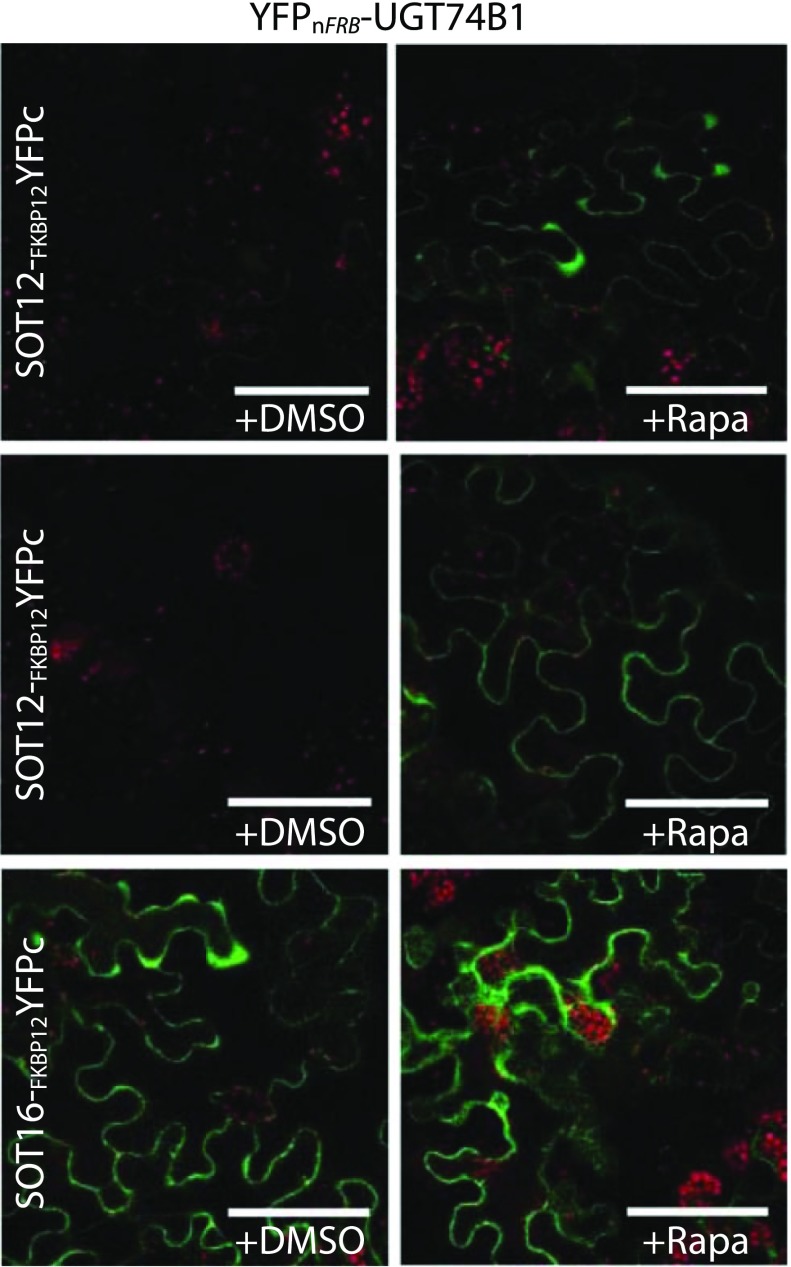
Application of chemically inducible built-in positive control in BiFC technique. Addition of rapamycin induces the interaction between the two proteins FRB and FKBP12. In the glucosinolate pathway, the interaction between the biosynthetic UGT74B1 and different SOT enzymes was investigated in *N. benthamiana* leaves by co-expressing either YFPn-FRB or YFPn-FRB-UGT74B1 with YFPc-FKBP12, SOT12-FKBP12-YFPc or SOT16-FKBP12-YFPc. Subsequently, the leaves were infiltrated with either water (+DMSO) or 30 μM rapamycin (+Rapa). The rapamycin-induced protein–protein interaction functions as a built-in positive control, to prove that the lack of interaction between the pair SOT12-UGT74B1 is not due to lack of expression and, to show the maximum fluorescence that can be obtained with the pair SOT16-UGT74B1. Scale bars represent 50 μm. (Courtesy of Scientific Reports, Andersen et al. 2016)

BiFC is a (relatively simple) method to monitor protein–protein interactions in vivo. In this method, a fluorescent protein is split into amino- and carboxy-terminal non-fluorescent fragments which are then fused to two proteins of interest. The BiFC assay is based on the association between two non-fluorescent fragments of a fluorescent protein when they are brought in proximity to each other by an interaction between proteins fused to the fragments. Once the fragmented fluorophore is reconstituted the complex is irreversible. BiFC provides information on the spatial localization (i.e. subcellular compartmentalization) of protein complexes (Kerppola 2008; Fig. 2). Although the irreversibility offers an advantage in detecting transient or weak interactions, it limits the use of BiFC assay for dynamic interactions. Furthermore, it opens up for false positive interactions i.e. the random collision of two proteins expressed in the same subcellular compartment. Proper negative controls in which mutations are introduced into the interaction interface in one of the two proteins may solve this problem (Kodama and Hu 2012). Additionally, BiFC assays are complicated in plants due to auto-fluorescence from the cell wall, chloroplasts and other cellular structures (Chen et al. 2008). Alternative methods have been developed using protein fragment complementation coupled to enzymatic assays such as firefly luciferase to measure protein–protein interactions (Fujikawa and Kato 2007; Chen et al. 2008). The luciferase-based complementation imaging assay requires the addition of the fluorescence-generating luciferin substrate. In contrast to BiFC, the luciferase-based complementation imaging enables measurement of dynamic nanometer scale protein–protein interactions and because the luminescence is measured in the dark, it is not affected by auto-fluorescence and thus is particularly attractive for plant studies (Chen et al. 2008).

## Fluorescence correlation spectroscopy (FCS) and fluorescence cross correlation spectroscopy (FCCS)

**Fig. 3:**
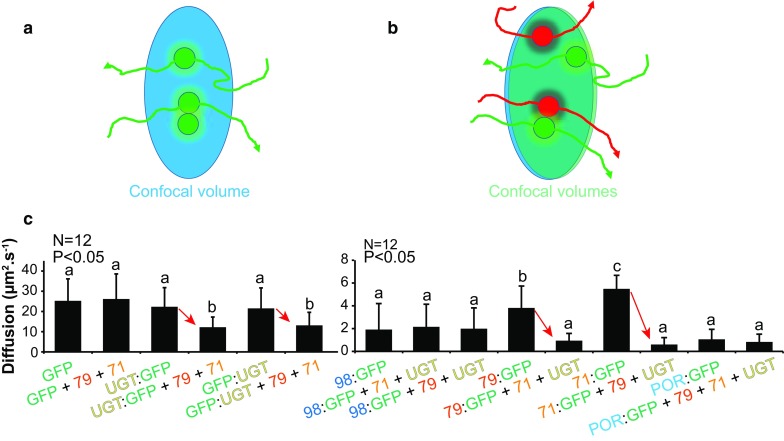
The principle of FCS and FCCS and application of FCS on the cyanogenic glucoside pathway. **a** FCS is used to show diffusion or determine local concentration of tagged target protein. All molecules of a specific target protein passing through the confocal volume are recorded to determine the autocorrelation curve. *Arrows* are indicating trajectories of molecules. **b** FCCS is used to show that two target proteins tagged with two different fluorophores can interact by following their co-trajectories. All molecules of the two target proteins passing through the confocal volume are recorded to determine the cross-correlation curve. *Arrows* are indicating trajectories of molecules. **c** The diffusion of proteins (CYP71E1, CYP79A1, UGT85B1) in dhurrin pathway was investigated by *in planta* FCS in *N. benthamiana* leaf epidermal cells transiently expressing GFP-tagged target proteins. GFP and CYP98A1 were used as controls. *Letters* indicate statistically significant similarities for the recorded values of the *t* test pairwise comparison with *p* < 0.05. *Error bars* indicate ±SD. *Red arrows* highlight the change of apparent diffusion constant. The apparent average diffusion constant of each partner (71, 79, UGT) was significantly lower when co-expressed with all its partners. These data supported the formation of a dynamic metabolon harboring the enzyme components catalyzing dhurrin synthesis. Interestingly, the P450-supporting reductase (POR) was not affected by co-expression of the dhurrin enzymes. (Courtesy of Science, Laursen et al. 2016)

FCS technique was developed in the early 1970s. FCS is a commonly used method, but still poorly exploited in plant science. The technique was first used for in vitro studies of diffusion of labeled macromolecules in solution and has recently been available for in vivo studies including *in planta* systems (Li et al. 2016). FCS monitors fluctuations in fluorescence emission from a target molecule (typically a fluorophore-tagged protein) due to movement of a population of this molecule in and out of a small defined confocal volume (approx. 0.25–0.5 fL) over time, as schematized in Fig. 3a with the green dots trajectories passing through the confocal volume. To estimate the crucial physical parameters of interest, the experimental autocorrelation function G(t) obtained by FCS is fitted with mathematical models for diffusion of fluorophore-tagged proteins. These models take into account (1) the size and shape of the confocal volume, (2) the excitation profile and the molecular brightness of the fluorophore, and (3) local concentrations, dynamic properties (diffusion, active transport), interactions or oligomerization states of target protein (Li et al. 2016). These physical parameters can in principle be determined in one recording, thus providing valuable information on the target protein (concentration, diffusion speed and aggregation state). The related FCCS technique monitors fluctuations in the fluorescence emission of at least two distinct fluorescent labels that can be individually excited and detected (Fig. 3b). Correlation of signals fluctuations of the fluorophores in the detection volume indicates co-diffusion, and thus association of the proteins at the single molecule level (Fig. 3b).

Despite the theoretical power of these methods, they have not been extensively applied *in planta* to date. Few important limitations may explain this under-utilization. The methods require sophisticated and still expensive equipment and software, and are complicated to implement. The methods rely on the precise measurements of the confocal volume, used for the calculations and the fitting to mathematical models. In living plant cells, the confocal volume measurement can be affected by the laser passing through the cell wall and plant tissues. The acquisition has to be achieved over sufficient amount of time to determine the autocorrelation function G(t), i.e. over many autocorrelations and from an averaging of thousands of molecule movements. Thus, it is highly challenging to target structures (proteins or metabolons) in a moving organelle, e.g. the ER, during long acquisition time. In Laursen et al. (2016), more than half of the recordings were discarded when targeted ER, or ER peripheral cytoplasm, had moved out of the focus plane (Bassard unpublished data). Accordingly, the cellular structures being observed must be immobile. In addition, FCS techniques are highly sensitive and require low concentrations of the fluorophore in a range of 0.1–100 nM (Li et al. 2016). Measurement of slow-diffusing molecules (<0.1 µm s^−1^, depending of the FCS equipment) is also difficult. Although in theory it is possible to deconvolute the data to highlight the different populations of diffusing molecules, it is challenging to do so *in planta*.

The different requirements limit the application of FCS and FCCS in living plant cells, where fluorophore concentrations, structure dynamics and background signals are not easy to control. However, FCS has been used in plant research to study protein dynamics (Goedhart et al. 2000; Köhler et al. 2000), to determine local concentration of target proteins (Li et al. 2011, 2013), and to monitor protein–protein interaction directly (Aker et al. 2007; Wang et al. 2013; Clark et al. 2016). Laursen et al. (2016) measured, in *Nicotiana benthamiana* expression system, diffusions of ER-associated proteins of the dhurrin pathway alone or co-expressed with their partner proteins. A reduction of the diffusion speed of each metabolon component, when associated to their partners, was observed and thus demonstrated association of each partner to large structures, which points to formation of the dhurrin metabolon (Fig. 3c). This kind of experiments using the FCS apparatus is very time-demanding. Here, approximately 90 h were spent, though without taking into account the time needed for the first optimizations of the procedure, and for preparing the materials (fusion constructs, Agrobacteria, plants).

New technological development constantly expands FCS possibilities. For example, the combination of Stimulated Emission Depletion (STED) microscopy and FCS pushed the *x*–*y* axis spatial resolution of FCS to 20–30 nm instead of 200 nm (Li et al. 2016). Generally, these new FCS implementations quickly become commercially available, and thus accessible to more researchers. FCS-based approaches are likely to be popular in quantitative analysis of single protein or metabolon in future plant research.

## Fluorescence resonance energy transfer (FRET)-based techniques

Co-localization experiments with two fluorescent fusion proteins observed with confocal microscopy are not enough to prove metabolon formation. The spatial resolution of the fluorescence microscopy is limited by the light diffraction (≈200 nm), and thus co-localization indicates co-occurrence in the confocal volume (200 by 200 by 600 nm) but not interaction. A possibility for going beyond the optical diffraction limit is to use new advanced methods like Single Molecule Localization Microscopy, but using two different fluorophores variants is challenging if not compatible with these methods. Over the last 15 years, in vivo detection of protein–protein interactions has become feasible by combining fluorescence microscopy and FRET-based techniques. These techniques are increasingly being used in plant research.

### The FRET principle

**Fig. 4:**
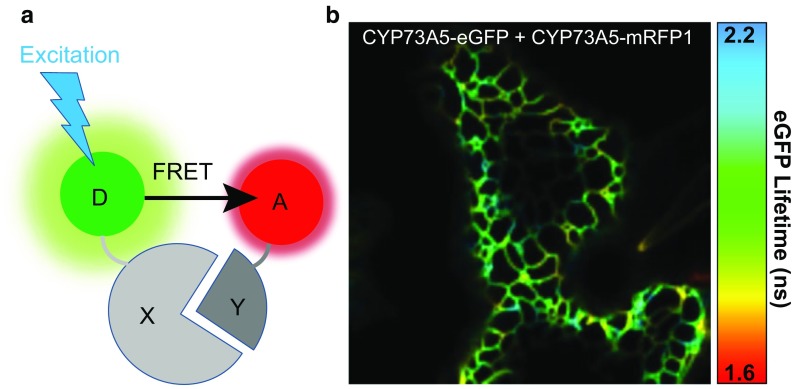
The principle in FRET-based techniques. **a** The FRET principle for protein–protein interaction between protein “*X*” and protein “*Y*” upon excitation of donor fluorophore “*D*”. If distance and orientation of the FRET donor “*D*” and acceptor “*A*” are acceptable, FRET will occur from *D* to *A*, when *D* is excited. **b** Pseudo-colored image showing lifetime spatial distribution. The image displays CYP73A5-eGFP fusion protein transiently co-expressed with CYP73A5-mRFP1 fusion protein in *N. benthamiana* leaf epidermal cell. Part of the cortical ER is visible and variation of lifetimes is measured across the ER, indicating subtle local heterogeneity in interaction of both CYP73A5 fusion proteins

To observe FRET, we need two fluorophores with a significant spectral overlap. FRET is based on a dipole–dipole resonance interaction that does not involve any light emission and absorption and in which non-radiative energy is transferred from an excited fluorescent molecule serving as a “donor” to another fluorescent molecule, the “acceptor” (Fig. 4a). With appropriate orientation of the fluorophores, FRET is occurring over a range of 1–10 nm (Gadella et al. 1999). The energy transfer leads to quenching in the fluorescence emission and to reduced lifetime of the donor, concomitantly FRET increases photon emission from the acceptor. FRET-based techniques are unique methods to monitor the functional dynamic changes of (1) biochemical activities, (2) conformation, and (3) particularly transient protein–protein interactions both in vitro and in vivo. Nevertheless, it must be emphasized that FRET techniques do not detect directly the interaction of the two tagged proteins, but the distance between the two fluorescent tags—a distance in the scale at which protein–protein interactions take place. Different methods are able to quantify FRET, each with distinct advantages and disadvantages. The methods have been extensively reviewed elsewhere (Bücherl et al. 2010; Sun et al. 2011; Becker 2012; Sun et al. 2013; Horvath et al. 2016; Tunc-ozdemir et al. 2016). With the sensitized FRET technique (Sun et al. 2013), FRET occurrence is measured by the increase in the photon emission of the acceptor in presence of the donor. The acceptor photobleaching FRET technique is based on the measurement of the donor recovery after acceptor photobleaching (Sun et al. 2013). These two techniques will briefly be reviewed although they have not been used for the study of our model biosynthetic pathways. Finally, the FLIM-based FRET measurements follow the change of donor fluorescence lifetime decay in presence of the acceptor.

### Acceptor photobleaching FRET and sensitized FRET techniques

The major problem associated with intensity measurement methodologies is to determine the spectral bleed-through that is detected in the FRET channel. The spectral bleed-through results from direct excitation of the acceptor by the donor excitation light or from donor emission signal that bleeds into the FRET detection channel. Accurate measurement of FRET requires correction methods that define and remove these different background components via several scans of the same sample prior to estimating the FRET efficiency. Thus, these techniques are difficult to apply to living cells and to dynamic metabolons. The acceptor photobleaching method is quite popular for measurement of FRET but it is usually applied to fixed cells or perfectly immobile environments, because the sample has to be scanned at least two times, before and after photobleaching (Poulsen et al. 2013). Sensitized emission FRET is not as easy to obtain as acceptor photobleaching FRET, but it gives much more consistent output with immobile samples (Tunc-ozdemir et al. 2016). The results obtained using fluorescence intensity measurements techniques are relative (Padilla-Parra and Tramier 2012), therefore results should be interpreted with caution. In addition, both techniques are sensitive to photobleaching of donor or acceptor, and are affected by plant cell auto-fluorescence. The advantage of these FRET techniques is that they are cheap to implement in any confocal or wide-field microscope. Thus, despite important limitations, these FRET-based techniques have been used successfully to study rather strong protein–protein interactions *in planta* (Kierzkowski et al. 2009).

### Fluorescence lifetime imaging microscopy (FLIM)

In recent years, FRET—measured by FLIM methods—has become the method of choice to probe and quantify protein–protein interactions in living cells. Fluorescence lifetime is the average time that a molecule spends in an excited state before returning to the ground state, typically with the emission of photons. FRET efficiency is precisely calculated by measuring the donor lifetime in the presence and the absence of acceptor. The donor lifetime is always shorter in the presence of an acceptor. Fluorescence lifetime measurements are implemented in wide-field, confocal, and multi-photon excitation microscopes, and determined in either the time domain or the frequency domain methods. The physics underneath these two different methods is identical, only the analysis of the measurements differs (Clegg 2010). The most accurate, the most employed and also the most time-consuming method—the time domain FLIM—can be measured by time-correlated single photon counting. In brief, the sample is excited with a pulsed laser source. The laser is synchronized to high-speed detectors and for each pulse the time between the excitation and the first detected emission photon is recorded (Sun et al. 2011). The photons are collected at different times to generate a decay curve and calculate the lifetime of the donor by fitting the experimental decay curve to a model decay curve. The frequency domain method uses a light source modulated at high radio frequencies to excite the FRET donor and then measure the change in the modulation and phase of the emission signals to extract the fluorescence lifetime (Sun et al. 2012). For more details on the two methods, the protocols and on comparisons of the two methodologies, see elsewhere (Gratton et al. 2003; Osterlund et al. 2015; Sun and Periasamy 2015; Padilla-Parra et al. 2015). Gratton et al. (2003) found that the signal-to-noise ratio of the lifetimes obtained for low-concentration donor (low photon count rates) is better for time domain FLIM than for frequency domain FLIM. At high concentration (high photon count rates) the signal-to-noise ratio of both techniques converges.

Prior to starting FLIM experiments, there are few important points to consider. First, it is necessary to acquire enough photon counts to reduce fitting ambiguities and obtain reliable calculation of lifetimes (Padilla-Parra et al. 2015). After data acquisition—in case the number of photons is too low for quantitative analysis—a binning factor can be used. Binning is a procedure where the selected pixel is analyzed, but the neighboring pixels are included for calculation of the fluorescence lifetime. Nevertheless, if the sample is sufficiently immobilized, it is preferred to increase acquisition times. Using a high-numerical-aperture 60× objective, a typical acquisition of an eGFP-labeled protein *in planta* cell takes 1–5 min, depending on the expression level of the eGFP-labeled target and the apparatus. By increasing the time of exposure, photobleaching in combination with low fluorophore abundance could be a problem (Becker 2012).

Secondly, FLIM methods are prone to false negative results. FRET cannot be detected in certain situations even if the target proteins interact to some extent. More often the amount of interacting donor per pixel is very low in comparison to the non-interacting donor, thus making it difficult to get a significant FRET signal. In this situation, fast acquisition FLIM should be performed to avoid the averaging of the FRET signals in space and time (Padilla-Parra et al. 2015). Using living cells and transient expression systems, this situation is difficult to control but can be contained by checking global expression levels of the tagged target proteins and by using the highest expressed protein fused to the FRET acceptor. For example, it was repeatedly observed that tagged soluble proteins were higher expressed than tagged ER-bound cytochromes P450 (Bassard et al. 2012; Laursen et al. 2016).

Thirdly, although the FLIM measurements are remarkably robust, heterogeneity in the measurements done *in planta* can be observed. One confocal image (or even one pixel) is a snapshot of all possible configurations between donor-tagged and acceptor-tagged targets. As the lifetime of a fluorescent molecule is sensitive to its local microenvironment, changes of pH and ions, could influence FRET determination, and thus this must be considered when comparing the lifetime distributions in different regions of a cell or tissue. Additionally, multiple lifetimes components can be detected *in planta* from auto-fluorescence background of several dyes. This auto-fluorescence exhibits decay constants in the same region (2–4 ns) as the commonly used fluorescence markers (Schleifenbaum et al. 2010). It is possible to determine the auto-fluorescence components using unlabeled samples (or alternatively use proper band-pass filters) to restrict the amount of dyes that are sampled by FLIM apparatus. If more exponential terms are necessary for the curve-fitting procedure, more photon counts are necessary in each pixel to achieve an accurate curve fitting. FLIM is able to differentiate subpopulations of lifetimes, but the data must be collected and analyzed from multiple cells to prevent the user from reaching false conclusions. Lastly, FLIM detection methods are limited by the cost of the apparatus and the sophistication of the analysis.

A major advantage of FLIM-based FRET measurements is that only the donor fluorescence decay needs to be measured, and the acquisition has to be done only one time per sample. In contrary to other FRET-based methods, FLIM methods are fluorophore concentrations-independent and not sensitive to (1) interference by spectral bleed-through, (2) change of excitation intensity, (3) some extent of photobleaching or light scattering, and finally to (4) the light path or the instrument employed (Lalonde et al. 2008; Becker 2012; Sun et al. 2012; Sun and Periasamy 2015; Horvath et al. 2016). Accordingly, FLIM is one of the most robust, most sensitive and most accurate FRET-based methods to study protein–protein interactions, even when protein concentration is not well-known, as is the case with *in planta* measurements (Bassard et al. 2012; Sun et al. 2015; Laursen et al. 2016). If multiple lifetimes components are present in the sample, FLIM methods are able to differentiate the subpopulations and to estimate the percentage of “FRETing” and “non-FRETing” donor populations. Nevertheless, it is recommendable to have a larger acceptor population than that of the donor (see limitations above). The FLIM method is well suited to determine the spatial and temporal distribution of interacting or closely associated proteins in living plant cells, and inform about the localization of metabolon formation (Fig. 4b). When experiments are done in vivo, one could test factors acting on the interaction, for example treat the cells with elicitors, hormones, stresses (Bassard et al. 2012), untagged partner proteins (Laursen et al. 2016), as well as use mutant plant lines to screen for interactions that may be dependent on such conditions (Wanke et al. 2011). The popular *Nicotiana benthamiana* transient expression system is very suitable for application of FLIM techniques due to the quick testing of multiple combinations of protein partners. In Laursen et al. 2016, 147 combinations were analyzed via FLIM method, including 74 negative or positive controls to cover all possible pairwise interactions between the four proteins of the dhurrin metabolon. All these combinations have been recorded over approximately 425 h using the FLIM apparatus, but without taking into account the time for the preliminary procedure optimizations, and for the preparation of the materials (fusion constructs, Agrobacteria and plants).

In conclusion, FLIM methods are particularly suited for in vivo studies of stable complexes and metabolons. FLIM methods are non-invasive and allow for the observation of protein–protein interactions almost in real time.

### Advantages and limitations of the different FRET-based techniques

It is tempting to calculate the distance between two target proteins from FRET values. However, the position of fluorophores on the target proteins must be known to calculate the distance. Extra cautions should be taken as the user cannot control the exact orientation of the fluorescent tags to the dipole–dipole orientation of the fluorophores (see above). An identical distance between two interacting protein targets with different dipole–dipole orientations of the fluorophores could give different FRET efficiencies, and even negative results (Vogel et al. 2006). Large interacting target proteins could also give negative results, if the distance between the two fluorophores is over 10 nm (depending of the fluorophore couple) (Dixit et al. 2006; Padilla-Parra et al. 2015). FRET measurements in vivo produce a snapshot of various individual configurations, and a change in average FRET value between different experimental conditions (stress, hormone treatments, etc.) could be explained by (1) a change of distance between target proteins, (2) a change of proportion of protein interacting, and (3) a combination of these two conditions. Thus, it is wise to have the most abundant interactant fused to the acceptor fluorophore to increase the likelihood of interaction of the donor with its partner (see above).

It is difficult to predict how many molecules are needed to interact to detect a signal in fluorescent-based techniques. Important factors that impact the level of fluorescence are sensitivity of the microscope, specimen background fluorescence, protein expression level and selected fluorophores. Furthermore, for *in planta* experiments, it is particularly difficult to anticipate the output and therefore to estimate the minimal number of molecules needed to detect fluorescence (it could be ten or thousands). Noticeable, specific in vitro setups are amenable to go to single molecule FRET detection (Bavishi and Hatzakis 2014).

An important point to be considered is the reproducibility of the in vivo FRET values. The data must be collected and statistically analyzed from multiple cells to prevent any false conclusions from non-representative measurements. Good positive and negative controls are fundamental to distinguish real protein interactions and random proximity in constrained environment such as e.g. the ER membrane. Furthermore, both lifetime and fluorescence intensity FRET techniques are temperature-dependent and only controlled environmental conditions will be reproducible (Osterlund et al. 2015). Finally, since many protein–protein interactions and protein activities have been discovered when associated with specific cellular components (Laursen et al. 2015), study of these proteins may only be considered within the context of the intact cell. Therefore, the development of non-invasive quantitative imaging techniques to visualize protein interactions inside living cells is essential for mapping the interactome. *In planta* studies provide spatio-temporal information that would not be possible to obtain using conventional biochemical or genetic methods.

## Considerations upon expression of (fluorophore-)tagged proteins

The tag-based techniques require tagged variants of the protein(s) of interest. The choice of the tag—as well as the way to attach it to the target protein—is critical. Some proteins might require a free N- or C-terminus to assure correct targeting, expression, functional activity, stability, mobility or interaction. We strongly recommend that all possible pairwise combinations of N- or C-terminally tagged proteins are tested, except if known membrane anchor or targeting signals are present. The use of linker between the tag and the protein of interest may help but the length and/or amino acid sequence of such linker have been shown to influence in an unpredictable manner inter- and intra-molecular FRET efficiencies either positively or negatively (Arai et al. 2001; Lissandron et al. 2005; Bhat et al. 2006). For in vivo experiments, the expression is often driven by strong constitutive promoters (e.g. the Cauliflower Mosaic Virus 35S promoter), which could result in ectopic expression and/or too high expression of the tagged proteins. This might subsequently result in artifacts that could possibly either promote or inhibit protein–protein interactions. Thus, wherever possible, expression must be checked or the native gene promoters should be used to drive the expression of tagged protein. In in vivo studies, endogenous and tagged proteins may compete for interaction partners, and thus possibly reduce the apparent FRET efficiencies (Dixit et al. 2006). Crosstalk between endogenous and inserted biosynthetic pathways might also be observed. Upon transient engineering of the dhurrin P450s into *Nicotiana benthamiana,* endogenous UGT(s) were able to convert the intermediate cyanohydrin to dhurrin, thus competing with the exogenous UGT85B1 (Laursen et al. 2016). However, reconstitution of the entire dhurrin metabolon in *N. benthamiana* host resulted in efficient production of dhurrin by tagged *Sorghum bicolor* enzymes (Laursen et al. 2016). In contrary, in a similarly designed study expressing tagged *S. bicolor* enzymes in *A. thaliana* host, no dhurrin production was observed (Nielsen et al. 2008). This suggests that factors related to the host can influence the outcome of in vivo studies.

## New tools for built-in positive controls in protein–protein interaction studies

When investigating interactions between two proteins with complementary reporter tags in yeast-two-hybrid or split GFP assays, it remains troublesome to discriminate true- from false-negative results and challenging to compare the level of interaction across experiments. This leads to decreased sensitivity and renders analysis of weak or transient interactions difficult to perform. A new tool was developed (Andersen et al. 2016), where reporters can be chemically induced with rapamycin to dimerize the FKBP12 and the FRB domains independently of the investigated interactions and thus alleviates false negatives. The chemically-induced dimerization serves as a built-in positive internal control. Thereby many of the drawbacks associated with evaluation of protein–protein interaction between two proteins of interest are overcome. For yeast and *in planta* work, the reporters have been incorporated into the widely used split ubiquitin-, BiFC- (Fig. 2) and FRET-based methods. The functionality of this concept has been demonstrated by the analysis of weakly interacting proteins from glucosinolate biosynthetic pathway in the model plant *A. thaliana*. UGT74B1 constructs and different SOT constructs were investigated in *N. benthamiana* leaves combining BiFC and rapamycin-induced protein–protein interaction, to prove that the lack of interaction between the pair SOT12-UGT74B1 is not due to lack of expression and, to show the maximum fluorescence that can be obtained with the pair SOT16-UGT74B1. The results illustrate that rapamycin-induced dimerization can function as a built-in control for split-based systems that is easily implemented and allows for direct evaluation of functionality.

## Future methodological advances

Theoretically, FRET technologies make it possible to perform single molecule experiments, depending on detector sensitivity and on the samples, and only for in vitro setup to date. The use of multiphoton excitation combined with FLIM system, particularly for plant cells, provides further advantages such as reduced phototoxicity and photobleaching (Schoberer and Botchway 2014) and better penetration into tissue. Automatization of the acquisition is now possible through (1) improvements in microscopy and software, (2) use of cell suspension cultures, multiwell plates and robots, and (3) advanced software for automatic cell detection and focus (Guzmán et al. 2016; Margineanu et al. 2016). FRET is also measurable with a flow cytometer (Hovarth et al. 2016). These improvements enable the use of an increased number of pairwise combinations, to test the interactome in the cell and to increase confidence in results.

Several promising methodologies are being developed or advanced, which will enable more precise studies of the dynamic and transient metabolons. Advanced mass spectrometry allows now the identification of intact, stable protein complexes (Hopper et al. 2013) and associated essential lipids (Laganowsky et al. 2014; Gault et al. 2016). Furthermore, temporal and spatial resolution improvement of microscopy-based approaches allows direct observations of the dynamic assembly processes in specific experimental conditions (Martinière et al. 2012; Hosy et al. 2015; Shi et al. 2016).

## Conclusion and perspectives on studying dynamic metabolons

We have reviewed several techniques that have been applied to study the existence of cyanogenic glucoside and glucosinolate metabolons. Studies on the cyanogenic pathway have focused on providing evidence for the interaction amongst the known players using advanced methods such as FCS and FRET-based technologies (Laursen et al. 2016), whereas the studies on the glucosinolate pathway have focused on untargeted screens using yeast-two-hybrid and Co-IP to identify the protein network surrounding the known players. In the latter study, no overlap was observed between the candidate lists generated by yeast-two-hybrid and Co-IP. Each method preferentially identified proteins with specific properties with respect to isoelectric point, hydrophilicity, length and transmembrane domains, with the yeast-two-hybrid approach identifying preferentially short, positively charged and membrane-bound candidates (Nintemann et al. unpubl. res.). Interestingly, the two methods identified proteins in distinct subnetworks, and with substantially interconnection between these subnetworks (Nintemann et al. unpubl. res.).

Highly dynamic structures as the metabolons cannot be described and characterized using single parameter analyses and one technique. For example, in case of involvement of homo- or hetero-oligomerizations, no information on the aggregation state could be recovered from FRET values, but the same fluorescent constructs could be used for FCS in a similar experimental setup to unravel the aggregation state. There are several examples of studies using distinct complementary techniques: FLIM and BiFC (Delporte et al. 2014), Co-IP and FLIM (Kriechbaumer et al. 2015), TAP-tag and yeast-2-hybrid (Goossens et al. 2016). It is feasible to combine BiFC- and FRET-based techniques by measuring FRET between multicolor BiFC constructs and thus testing interactions for four proteins (Kwaaitaal et al. 2010). Moreover, BiFC has been used with flow cytometry in Berendzen et al. 2012, for fast screening of 6393 positive BIFC signals, to finally identify eight new interactors. Multimodal fluorescence image spectroscopy techniques (Weidtkamp-Peters et al. 2009; Levitt et al. 2015) or Fluorescence Lifetime Correlation Spectroscopy (Chen and Irudayaraj 2010) can reveal more physical parameters of proteins than separate FCS or FRET-based techniques. All these combinations of approaches not only help to understand the multiple facets of metabolon formation, but also deal with false negative and false positive results that are specific to each approach.

Metabolons are transient assemblies, relying on delicate local changes in solutes, structural elements and possibly scaffolding proteins (Laursen et al. 2015; Dastmalchi and Facchini 2016; Bassard et al. 2017). Transient interactions between proteins participating in metabolon formation inside living cells are fundamental to many cellular processes. Traditionally, the yeast-2-hybrid or Co-IP approaches represent the methods of choice to discover protein–protein interaction networks on a large and high-throughput scale, and as described above these methods are not suitable for transient interactions. The continued development of non-invasive quantitative imaging techniques (FCS, FLIM) is critical for visualization of protein–protein interactions inside intact plant cells. In conclusion, only the combination of targeted and untargeted approaches allows for a better understanding of the orchestration of metabolons within the protein network in which they are imbedded.

Despite significant progress in metabolon research, several parameters characterizing metabolons are still poorly understood or overlooked e.g. how are compartment-spanning biosynthetic pathways orchestrated across different organelles and/or cells? How are metabolites (precursors, intermediates, end products, solvents) distributed? And can these metabolites guide metabolon formation? Ultimately, the knowledge obtained by unraveling the mechanisms regulating metabolon formation and metabolic flux will enable predictable transplant of plant biosynthetic pathways into heterologous host for production of high value bioactive natural products via synthetic biology approaches (Dueber et al. 2009; Farré et al. 2014; Singleton et al. 2014).

## Abbreviations

BiFC: Bimolecular fluorescence complementation
Co-IP: Co-immunoprecipitation
ER: Endoplasmic reticulum
FCS: Fluorescence correlation spectroscopy
FCCS: Fluorescence cross-correlation spectroscopy
FLIM: Fluorescence lifetime imaging microscopy
FRET: Fluorescence/Förster Resonance Energy Transfer
GGP1: γ-glutamyl peptidase 1
GFP: Green fluorescent protein
GST: Glutathione S-transferase
MS: Mass spectrometry
P450: Cytochrome P450
STED: Stimulated emission depletion microscopy
SOT: Sulfotransferase
TAP: Tandem affinity purification
UGT: UDP-glucosyl transferase
Y2H: Yeast-2-hybrid

## References

[CR1] Aker J, Hesselink R, Engel R (2007). In vivo hexamerization and characterization of the *Arabidopsis* AAA ATPase CDC48A complex using forster resonance energy transfer-fluorescence lifetime imaging microscopy and fluorescence correlation spectroscopy. Plant Physiol.

[CR2] An S, Kumar R, Sheets ED (2008). Reversible compartmentalization of de novo purine biosynthetic complexes in living cells. Science.

[CR3] Andersen TG (2012) Novel molecular tools for crop development and synthesis of natural products—understanding glucosinolate based defenses in cruciferous plants. Ph.D. dissertation, University of Copenhagen

[CR4] Andersen TG, Nintemann SJ, Marek M (2016). Improving analytical methods for protein–protein interaction through implementation of chemically inducible dimerization. Sci Rep.

[CR5] Andrès C, Agne B, Kessler F (2011). Preparation of multiprotein complexes from *Arabidopsis chloroplasts* using tandem affinity purification. Methods Mol Biol.

[CR6] Arai R, Ueda H, Kitayama A (2001). Design of the linkers which effectively separate domains of a bifunctional fusion protein. Protein Eng.

[CR7] Bassard JE, Richert L, Geerinck J (2012). Protein–protein and protein–membrane associations in the lignin pathway. Plant Cell.

[CR8] Bassard JE, Møller BL, Laursen T (2017). Assembly of dynamic P450-mediated metabolons—order versus chaos. Curr Mol Biol Rep.

[CR9] Bavishi K, Hatzakis NS (2014). Shedding light on protein folding, structural and functional dynamics by single molecule studies. Molecules.

[CR10] Becker W (2012). Fluorescence lifetime imaging–techniques and applications. J Microsc.

[CR11] Berendzen KW, Böhmer M, Wallmeroth N (2012). Screening for in planta protein–protein interactions combining bimolecular fluorescence complementation with flow cytometry. Plant Methods.

[CR12] Bhat RA, Lahaye T, Panstruga R (2006). The visible touch: in planta visualization of protein–protein interactions by fluorophore-based methods. Plant Methods.

[CR13] Braun P, Aubourg S, Van Leene J (2013). Plant protein interactomes. Annu Rev Plant Biol.

[CR14] Bücherl C, Aker J, de Vries S (2010). Probing protein–protein interactions with FRET-FLIM methods. Mol Biol.

[CR15] Castellana M, Wilson MZ, Xu Y (2014). Enzyme clustering accelerates processing of intermediates through metabolic channeling. Nat Biotechnol.

[CR16] Chen J, Irudayaraj J (2010). Fluorescence lifetime cross correlation spectroscopy resolves EGFR and antagonist interaction in live cells. Anal Chem.

[CR17] Chen H, Zou Y, Shang Y (2008). Firefly luciferase complementation imaging assay for protein–protein interactions in plants. Plant Physiol.

[CR18] Chen HC, Li Q, Shuford CM (2011). Membrane protein complexes catalyze both 4- and 3-hydroxylation of cinnamic acid derivatives in monolignol biosynthesis. Proc Natl Acad Sci USA.

[CR19] Chen HC, Song J, Wang JP (2014). Systems biology of lignin biosynthesis in *Populus trichocarpa*: heteromeric 4-coumaric acid:coenzyme A ligase protein complex formation, regulation, and numerical modeling. Plant Cell.

[CR20] Clark NM, Hinde E, Winter CM (2016). Tracking transcription factor mobility and interaction in *Arabidopsis* roots with fluorescence correlation spectroscopy. Elife.

[CR21] Clegg RM, Clegg RM, Periasamy A (2010). Fluorescence lifetime-resolved imaging what, why, how—A prologue. FLIM microscopy in biology and medicine.

[CR22] Crosby KC, Pietraszewska-Bogiel A, Gadella TW (2011). Förster resonance energy transfer demonstrates a flavonoid metabolon in living plant cells that displays competitive interactions between enzymes. FEBS Lett.

[CR23] Dastmalchi M, Facchini PJ (2016). Plant metabolons assembled on demand. Science.

[CR24] Dastmalchi M, Bernards MA, Dhaubhadel S (2016). Twin anchors of the soybean isoflavonoid metabolon: evidence for tethering of the complex to the endoplasmic reticulum by IFS and C4H. Plant J.

[CR25] Deffar K, Shi H, Li L (2009). Nanobodies—the new concept in antibody engineering. Afr J Biotechnol.

[CR26] Delporte A, De Vos WH, Van Damme EJ (2014). In vivo interaction between the tobacco lectin and the core histone proteins. J Plant Physiol.

[CR27] Dixit R, Cyr R, Gilroy S (2006). Using intrinsically fluorescent proteins for plant cell imaging. Plant J.

[CR28] Dmitriev OY, Lutsenko S, Muyldermans S (2016). Nanobodies as probes for protein dynamics in vitro and in cells. J Biol Chem.

[CR29] Dueber JE, Wu GC, Malmirchegini GR (2009). Synthetic protein scaffolds provide modular control over metabolic flux. Nat Biotechnol.

[CR30] Farré G, Blancquaert D, Capell T (2014). Engineering complex metabolic pathways in plants. Annu Rev Plant Biol.

[CR31] Fields S, Song O (1989). A novel genetic system to detect protein–protein interactions. Nature.

[CR32] Fujikawa Y, Kato N (2007). Split luciferase complementation assay to study protein–protein interactions in *Arabidopsis* protoplasts. Plant J..

[CR33] Gadella TW, van der Krogt GN, Bisseling T (1999). GFP-based FRET microscopy in living plant cells. Trends Plant Sci.

[CR34] Gault J, Donlan JA, Liko I (2016). High-resolution mass spectrometry of small molecules bound to membrane proteins. Nat Methods.

[CR35] Gerace E, Moazed D (2015). Affinity purification of protein complexes using TAP tags. Methods Enzymol.

[CR36] Goedhart J, Hink MA, Visser AJ (2000). In vivo fluorescence correlation microscopy (FCM) reveals accumulation and immobilization of Nod factors in root hair cell walls. Plant J.

[CR37] Goossens J, De Geyter N, Walton A (2016). Isolation of protein complexes from the model legume *Medicago truncatula* by tandem affinity purification in hairy root cultures. Plant J.

[CR38] Gratton E, Breusegem S, Sutin J (2003). Fluorescence lifetime imaging for the two-photon microscope: timedomain and frequency domain methods. J Biomed Opt.

[CR39] Guzmán C, Oetken-Lindholm C, Abankwa D (2016). Automated high-throughput fluorescence lifetime imaging microscopy to detect protein–protein interactions. J Lab Autom.

[CR40] Hengen PN (1997). False positives from the yeast two-hybrid system. Trends Biochem Sci.

[CR41] Hopper JT, Yu YT, Li D (2013). Detergent-free mass spectrometry of membrane protein complexes. Nat Methods.

[CR42] Horvath GL, Langhoff P, Latz E (2016). Toll-like receptor interactions measured by microscopic and flow cytometric FRET. Methods Mol Biol.

[CR43] Hosy E, Martinière A, Choquet D (2015). Super-resolved and dynamic imaging of membrane proteins in plant cells reveal contrasting kinetic profiles and multiple confinement mechanism. Mol Plant.

[CR44] Jørgensen K, Rasmussen AV, Morant M (2005). Metabolon formation and metabolic channeling in the biosynthesis of plant natural products. Curr Opin Plant Biol.

[CR45] Kerppola TK (2008). Bimolecular fluorescence complementation (BiFC) analysis as a probe of protein interactions in living cells. Annu Rev Biophys.

[CR46] Kierzkowski D, Kmieciak M, Piontek P (2009). The *Arabidopsis* CBP20 targets the cap-binding complex to the nucleus, and is stabilized by CBP80. Plant J.

[CR47] Kittanakom S, Chuk M, Wong V (2009). Analysis of membrane protein complexes using the split-ubiquitin membrane yeast two-hybrid (MYTH) system. Methods Mol Biol.

[CR48] Kodama Y, Hu CD (2012). Bimolecular fluorescence complementation (BiFC): a 5-year update and future perspectives. Biotechniques.

[CR49] Köhler RH, Schwille P, Webb WW (2000). Active protein transport through plastid tubules: velocity quantified by fluorescence correlation spectroscopy. J Cell Sci.

[CR50] Kriechbaumer V, Botchway SW, Slade SE (2015). Reticulomics: protein–protein interaction studies with two plasmodesmata-localized reticulon family proteins identify binding partners enriched at plasmodesmata, endoplasmic reticulum, and the plasma membrane. Plant Physiol.

[CR51] Kwaaitaal M, Keinath NF, Pajonk S (2010). Combined bimolecular fluorescence complementation and Forster resonance energy transfer reveals ternary SNARE complex formation in living plant cells. Plant Physiol.

[CR52] Kwiatkowska M, Polit JT, Stępiński D (2015). Lipotubuloids in ovary epidermis of *Ornithogalum umbellatum* act as metabolons: suggestion of the name ‘lipotubuloid metabolon’. J Exp Bot.

[CR53] Kyoung M, Russell SJ, Kohnhorst CL (2015). Dynamic architecture of the purinosome involved in human de novo purine biosynthesis. Biochemistry.

[CR54] Laganowsky A, Reading E, Allison TM (2014). Membrane proteins bind lipids selectively to modulate their structure and function. Nature.

[CR55] Lallemand B, Erhardt M, Heitz T (2013). Sporopollenin biosynthetic enzymes interact and constitute a metabolon localized to the endoplasmic reticulum of tapetum cells. Plant Physiol.

[CR56] Lalonde S, Ehrhardt DW, Loqué D (2008). Molecular and cellular approaches for the detection of protein–protein interactions: latest techniques and current limitations. Plant J.

[CR57] Laursen T, Møller BL, Bassard JE (2015). Plasticity of specialized metabolism as mediated by dynamic metabolons. Trends Plant Sci.

[CR58] Laursen T, Borch J, Knudsen C (2016). Characterization of a dynamic metabolon producing the defense compound dhurrin in sorghum. Science.

[CR59] Levitt JA, Morton PE, Fruhwirth GO (2015). Simultaneous FRAP, FLIM and FAIM for measurements of protein mobility and interaction in living cells. Biomed Opt Express.

[CR60] Li Y (2010). Commonly used tag combinations for tandem affinity purification. Biotechnol Appl Biochem.

[CR61] Li X, Wang X, Yang Y (2011). Single-molecule analysis of PIP2;1 dynamics and partitioning reveals multiple modes of *Arabidopsis* plasma membrane aquaporin regulation. Plant Cell.

[CR62] Li X, Luu DT, Maurel C (2013). Probing plasma membrane dynamics at the single-molecule level. Trends Plant Sci.

[CR63] Li X, Xing J, Qiu Z (2016). Quantification of membrane protein dynamics and interactions in plant cells by fluorescence correlation spectroscopy. Mol Plant.

[CR64] Lissandron V, Terrin A, Collini M (2005). Improvement of a FRET-based indicator for cAMP by linker design and stabilization of donor–acceptor interaction. J Mol Biol.

[CR65] Luu W, Hart-Smith G, Sharpe LJ (2015). The terminal enzymes of cholesterol synthesis, DHCR24 and DHCR7, interact physically and functionally. J Lipid Res.

[CR66] Margineanu A, Chan JJ, Kelly DJ (2016). Screening for protein–protein interactions using Förster resonance energy transfer (FRET) and fluorescence lifetime imaging microscopy (FLIM). Sci Rep.

[CR67] Marsh JA, Teichmann SA (2015). Structure, dynamics, assembly, and evolution of protein complexes. Annu Rev Biochem.

[CR68] Martinière A, Lavagi I, Nageswaran G (2012). Cell wall constrains lateral diffusion of plant plasma-membrane proteins. Proc Natl Acad Sci USA.

[CR69] Merkley ED, Cort JR, Adkins JN (2013). Cross-linking and mass spectrometry methodologies to facilitate structural biology: finding a path through the maze. J Struct Funct Genomics.

[CR70] Møller BL (2010). Dynamic metabolons. Science.

[CR71] Nallamilli BR, Zhang J, Mujahid H (2013). Polycomb group gene OsFIE2 regulates rice (Oryza sativa) seed development and grain filling via a mechanism distinct from *Arabidopsis*. PLoS Genet.

[CR72] Narayanaswamy R, Levy M, Tsechansky M (2009). Widespread reorganization of metabolic enzymes into reversible assemblies upon nutrient starvation. Proc Natl Acad Sci USA.

[CR73] Nielsen KA, Tattersall DB, Jones PR (2008). Metabolon formation in dhurrin biosynthesis. Phytochemistry.

[CR74] Nintemann (2016) The spatial organization of glucosinolate biosynthesis—localization of biosynthetic enzymes from the whole plant to the subcellular level. Ph.D. dissertation, University of Cpoenhagen

[CR75] Osterlund EJ, Liu Q, Andrews DW (2015). The use of FLIM-FRET for the detection of mitochondria-associated protein interactions. Methods Mol Biol.

[CR76] Padilla-Parra S, Tramier M (2012). FRET microscopy in the living cell: different approaches, strengths and weaknesses. BioEssays.

[CR77] Padilla-Parra S, Audugé N, Tramier M (2015). Time-domain fluorescence lifetime imaging microscopy: a quantitative method to follow transient protein-protein interactions in living cells. Cold Spring Harb Protoc.

[CR78] Petschnigg J, Wong V, Snider J (2012). Investigation of membrane protein interactions using the split-ubiquitin membrane yeast two-hybrid system. Methods Mol Biol.

[CR79] Poulsen CP, Vereb G, Geshi N, Schulz A (2013). Inhibition of cytoplasmic streaming by cytochalasin D is superior to paraformaldehyde fixation for measuring FRET between fluorescent protein-tagged Golgi components. Cytometry Part A.

[CR80] Ralston L, Yu O (2006). Metabolons involving plant cytochrome P450. Phytochem Rev.

[CR81] Rigaut G, Shevchenko A, Rutz B (1999). A generic protein purification method for protein complex characterization and proteome exploration. Nat Biotechnol.

[CR82] Rohila JS, Chen M, Cerny R (2004). Improved tandem affinity purification tag and methods for isolation of protein heterocomplexes from plants. Plant J.

[CR83] Schleifenbaum F, Elgass K, Sackrow M (2010). Fluorescence intensity decay shape analysis microscopy (FIDSAM) for quantitative and sensitive live cell imaging. Mol Plant.

[CR84] Schoberer J, Botchway SW (2014). Investigating protein-protein interactions in the plant endomembrane system using multiphoton-induced FRET-FLIM methods. Mol Biol.

[CR85] Shi X, Kohram M, Zhuang X (2016). Interactions and translational dynamics of Phosphatidylinositol Bisphosphate (PIP2) lipids in asymmetric lipid bilayers. Langmuir.

[CR86] Singleton C, Howard TP, Smirnoff N (2014). Synthetic metabolons for metabolic engineering. J Exp Bot.

[CR87] Sønderby IE, Geu-Flores F, Halkier BA (2010). Biosynthesis of glucosinolates–gene discovery and beyond. Trends Plant Sci.

[CR88] Speth C, Toledo-Filho LA, Laubinger S (2014). Immunoprecipitation-based analysis of protein–protein interactions. Methods Mol Biol.

[CR89] Srere PA (1985). The metabolon. Trends Biochem Sci.

[CR90] Stagljar I, Korostensky C, Johnsson N (1998). A genetic system based on split-ubiquitin for the analysis of interactions between membrane proteins in vivo. Proc Natl Acad Sci USA.

[CR92] Sun Y, Day RN, Periasamy A (2011). Investigating protein-protein interactions in living cells using fluorescence lifetime imaging microscopy. Nat Protoc.

[CR93] Sun Y, Hays NM, Periasamy A (2012). Monitoring protein interactions in living cells with fluorescence lifetime imaging microscopy. Methods Enzymol.

[CR94] Sun Y, Rombola C, Jyothikumar V (2013). Förster resonance energy transfer microscopy and spectroscopy for localizing protein–protein interactions in living cells. Cytometry A.

[CR91] Sun Y, Periasamy A (2015). Localizing protein–protein interactions in living cells using fluorescence lifetime imaging microscopy. Methods Mol Biol.

[CR95] Szecowka M, Heise R, Tohge T (2013). Metabolic fluxes in an illuminated *Arabidopsis rosette*. Plant Cell.

[CR96] Tunc-Ozdemir M, Fu Y, Jones AM (2016). Cautions in measuring in vivo interactions using FRET and BiFC in *Nicotiana benthamiana*. Methods Mol Biol.

[CR97] Van Leene J, Eeckhout D, Persiau G (2011). Isolation of transcription factor complexes from *Arabidopsis* cell suspension cultures by tandem affinity purification. Methods Mol Biol.

[CR98] Van Leene J, Blomme J, Kulkarni SR (2016). Functional characterization of the *Arabidopsis* transcription factor bZIP29 reveals its role in leaf and root development. J Exp Bot.

[CR99] Vogel SS, Thaler C, Koushik SV (2006). Fanciful FRET. Sci STKE.

[CR100] Wang Q, Zhao Y, Luo W (2013). Single-particle analysis reveals shutoff control of the Arabidopsis ammonium transporter AMT1;3 by clustering and internalization. Proc Natl Acad Sci USA.

[CR101] Wanke D, Hohenstatt ML, Dynowski M (2011). Alanine zipper-like coiled-coil domains are necessary for homotypic dimerization of plant GAGA-factors in the nucleus and nucleolus. PLoS ONE.

[CR102] Weidtkamp-Peters S, Felekyan S, Bleckmann A (2009). Multiparameter fluorescence image spectroscopy to study molecular interactions. Photochem Photobiol Sci.

[CR103] Weis C, Pfeilmeier S, Glawischnig E (2013). Co-immunoprecipitation-based identification of putative BAX INHIBITOR-1-interacting proteins involved in cell death regulation and plant-powdery mildew interactions. Mol Plant Pathol.

[CR104] Winkel BS (2004). Metabolic channeling in plants. Annu Rev Plant Biol.

[CR105] Wu F, Minteer S (2015). Krebs cycle metabolon: structural evidence of substrate channeling revealed by cross-linking and mass spectrometry. Angew Chem Int Ed Engl.

